# Genome-Wide Enhancer Analysis Reveals the Role of AP-1 Transcription Factor in Head and Neck Squamous Cell Carcinoma

**DOI:** 10.3389/fmolb.2021.701531

**Published:** 2021-08-02

**Authors:** Chen-Yu Wang, Guang-Tao Yu, Chuan Gao, Ji Chen, Qing-Lan Li, Lu Zhang, Min Wu, Zhi-Jun Sun, Lian-Yun Li

**Affiliations:** ^1^ Frontier Science Center for Immunology and Metabolism, College of Life Sciences, Hubei Key Laboratory of Cell Homeostasis, Hubei Key Laboratory of Developmentally Originated Disease, Hubei Key Laboratory of Enteropathy, Wuhan University, Wuhan, China; ^2^ The State Key Laboratory Breeding Base of Basic Science of Stomatology (Hubei-MOST) and Key Laboratory of Oral Biomedicine, Ministry of Education, School and Hospital of Stomatology, Wuhan University, Wuhan, China

**Keywords:** HNSCC, enhancer, H3K27ac, H3K4me1, AP-1

## Abstract

Head and neck squamous cell carcinoma (HNSCC) is one of the most common cancers in the world, but its epigenomic features have not been determined. Here, we studied the chromatin landscape of active enhancers of HNSCC head tumor tissues by performing H3K27ac and H3K4me1 ChIP-Seq with a *Tgfbr1/Pten* double conditional knockout HNSCC mouse model. We identified 1,248 gain variant enhancer loci (VELs) and 2,188 lost VELs, as well as 153 gain variant super enhancer loci (VSELs) and 234 lost VSELs. Potentially involved transcription factors were predicted with motif analysis, and we identified AP-1 as one of the critical oncogenic transcription factors in HNSCC and many other types of cancer. Combining transcriptomic and epigenomic data, our analysis also showed that AP-1 and histone modifications coordinately regulate target gene expression in HNSCC. In conclusion, our study provides important epigenomic information for enhancer studies in HNSCC and reveals new mechanism for AP-1 regulating HNSCC.

## Introduction

Head and neck squamous cell carcinoma (HNSCC) is one of the most common human malignancies in the world, with poor prognosis for patients (Leemans et al., 2018). Many studies have shown that epigenetic factors play critical roles in HNSCC development and progression, including DNA methylation, histone modifications, non-coding RNAs, and so on (Castilho et al., 2017; Leemans et al., 2018; Gazdzicka et al., 2020). Epigenetic abnormality promotes malignancy through repressing the expression of tumor suppressive genes, upregulating oncogenes, changing chromatin structure, or inducing genome instability (Yao et al., 2020). Further understanding of the epigenetic features of HNSCC will provide new biomarkers and novel strategies for diagnosis and treatment.

Enhancers are cis-acting elements for transcription factor (TF) binding, which activate gene transcription over a long distance (Calo and Wysocka, 2013; Shlyueva et al., 2014; Nizovtseva et al., 2017). It has been hypothesized that enhancer activity elevation is one of the common features of cancer cells, which was supported by several recent studies (Hnisz et al., 2013). However, the genome-wide landscape of active enhancers in HNSCC has not been studied yet. Specific patterns of histone modifications occupy the surrounding nucleosomes of enhancers, which are often used to identify novel enhancers. Usually, H3K4me1 is the basic mark to label enhancers. If H3K27ac is present, the enhancer is active; if H3K27me3 occupies the region instead of H3K27ac, the enhancer is poised (Shlyueva et al., 2014; Nizovtseva et al., 2017). Nowadays, ChIP-Seq of the above histone modifications is often used to annotate enhancers. One study on mouse embryonic stem cells identified 25,036 enhancers using chromatin regions enriched with H3K4me1 (Creyghton et al., 2010). Their results indicated H3K27ac as an active enhancer hallmark. H3K27ac and H3K4me3 are hallmarks for active promoters of expressed gene; thus, only H3K27ac-enriched regions far away from promoters are considered as active enhancers (Rickels and Shilatifard, 2018).

The correlation between enhancer activities and malignancy has been proposed by several groups (Hnisz et al., 2013; Pott and Lieb, 2015; Roe et al., 2017; Yuan et al., 2017). Interestingly, many histone-modifying enzymes involved in enhancer regulation are frequently mutated in cancer cells, such as lysine methyltransferase 2C/D (KMT2C/D, also known as MLL3/4) and E1A binding protein p300/CREB binding protein (p300/CBP), which are key enzymes for H3K4me1 and H3K27ac, respectively (Yao et al., 2020). MLL3 mutation in cells results in the defect of BRCA1-associated protein 1 (BAP1) recruitment to enhancers of tumor-suppressor genes, which represses enhancer activity and promotes tumorigenesis (Wang et al., 2018). In breast cancer, the defect of lysine demethylase 5C (KDM5C) causes elevation of H3K4me3 on oncogene enhancers, which activates oncogene expression and tumorigenesis (Shen et al., 2016). Generally, H3K4me3 marks gene transcription start sites (TSS) (Eissenberg and Shilatifard, 2010). However, evidence is accumulating that H3K4me3 also exists on a portion of overactive enhancers, whose functions are associated with tumorigenesis (Shen et al., 2016; Hu et al., 2017; Li et al., 2019). Moreover, the enhancer activity of target genes of specific cancer-related signaling pathways is regulated by epigenetic factors. For example, lysine demethylase 3A (KDM3A) removes H3K9me2 on enhancers and promotes the gene expression downstream of the hippo pathway (Wang et al., 2019). GLIS family zinc finger 2 (GLIS2), an oncogenic transcription factor, represses the enhancer activity of p53 target genes (Yao et al., 2020). Recently, the concept of super enhancers was raised, and it has been hypothesized that super enhancers of oncogenes are associated with tumorigenesis (Hnisz et al., 2013; Pott and Lieb, 2015). Therefore, it is critical to fully understand the roles and mechanisms of enhancer regulation in tumorigenesis and metastasis.

In the current study, to understand the epigenomic dynamics in HNSCC, we investigate the genome-wide landscape of active enhancers in an HNSCC mouse model using H3K4me1 and H3K27ac ChIP-Seq, which provides important mechanistic information for HNSCC.

## Materials and Methods

### Mice Feeding and Tissue Collection

All animal experiments were performed under the guidelines of the Animal Experimentations Ethics Committee of Wuhan University. *Tgfbr1/Pten* 2cKO mice was generated as previously described (Bian et al., 2012). *Tgfbr1* cKO mice (mixed genetic strains of FVB/N, C57BL/6, 129SV/J, and CD1) were crossed with the *Ptenflox/flox* mouse line (genetic strain 129SV/J) to generate mice heterozygous for both *Tgfbr1* flox and *Pten* foxp with *K14-CreERtam* (*K14-CreERtam;Tgfbr1f/+; Ptenf/+*). After five consecutive days of tamoxifen treatments by oral gavage, *Tgfbr1*/*Pten* was knocked out in the epithelium of the oral cavity and head-neck region. During induction, squamous cell carcinoma occurred in the head-neck region of the mice. This mouse model was maintained and genotyped as the previous description (Bian et al., 2012).

### Chromatin Immunoprecipitation Assay of Mouse Tissues

The ChIP assay was performed as previously described (Li et al., 2019; Yao et al., 2020). 60 mg of tissue was cut into 1 mm^3^ pieces in PBS with a protease inhibitor, cross-linked for 10 min at room temperature with 1% formaldehyde, and then quenched with 0.125 M glycine for 5 min. Cross-linked tissues were triturated by trituration equipment for 30 s and then centrifuged at 12,000 rpm, 4°C for 5 min. The precipitates were lysed with 1 ml lysis buffer (50 mM Tris-HCl pH 8.0, 0.1% SDS, and 5 mM EDTA) and incubated for 5 min with gentle rotation. After centrifugation at 12,000 rpm and 4°C for 2 min, lysates were washed once by digestion buffer (50 mM Tris-HCl pH 8.0, 1 mM CaCl_2_, and 0.2% Triton X-100). Washed lysates were incubated in 630 μL digestion buffer with 1 μl MNase (NEB, M0247S) at 37°C for 20 min and then quenched with 8 μl 0.5 M EDTA. Whole lysates were sonicated and the supernatants were taken out after centrifugation. 30 μl of supernatants were taken for checking MNase digestion efficiency. Antibodies for H3K27ac (Abcam Ab4729) and H3K4me1 (CST 5326) were purchased from the indicated merchants. Immunoprecipitation was performed with 150 μl sheared chromatin, 2 μg antibody, 50 μl Protein G Sepharose beads, and 800 μl dilution buffer (20 mM Tris-HCl pH 8.0, 150 mM NaCl, 2 mM EDTA, 1% Triton X-100, and 0.1% SDS) overnight at 4°C. Next day, immune-complexes were washed once with Wash buffer I (20 mM Tris-HCl pH 8.0, 150 mM NaCl, 2 mM EDTA, 1% Triton X-100, and 0.1% SDS), once with Wash buffer II (20 mM Tris-HCl pH 8.0, 500 mM NaCl, 2 mM EDTA, 1% Triton X-100, and 0.1% SDS), once with Wash buffer III (10 mM Tris-HCl pH 8.0, 250 mM LiCl, 1 mM EDTA, 1% Na-deoxycholate, and 1% NP-40), and twice with TE (10 mM Tris-HCl pH 8.0 and 1 mM EDTA). The immune-complexes were eluted twice with 100 μL elution buffer (1% SDS, 0.1M NaHCO_3_, and 20 mg/ml Proteinase K) at room temperature. The elution was incubated at 65°C for 6 h and then purified with a DNA purification kit (TIANGEN DP214-03).

### ChIP-Seq Library Construction

ChIP-seq libraries were constructed by using the VATHS Universal DNA Library Prep Kit for Illumina (Vazyme ND604). Briefly, 50 μL purified ChIP DNA (8–10 ng) was end-repaired for dA tailing, followed by adaptor ligation. Each adaptor was marked with a barcode of 6 bp, which can be recognized after mixing different samples together. Adaptor-ligated ChIP DNA was purified by AMPure XP beads (1:1) and then amplified by PCR for 11–13 cycles with the primer matching with the adaptor universal part. The amplified ChIP DNA was purified again using AMPure XP beads (1:1) in 35 μl EB elution buffer. For multiplexing, libraries with different barcodes were mixed together with equal molar quantities by considering appropriate sequencing depth (30–40 million reads per library). Libraries were sequenced by the Illumina Hi-seq X Ten platform with pair-end reads of 150 bp.

### RNA-Seq Library Construction

RNA was extracted using the EASYspin RNA Mini Kit (Aidlab RN07). 20 mg tissue was triturated by trituration equipment for 20 s in lysis buffer provided by the kit and then centrifuged at 12,000 rpm, 4°C for 5 min. The liquid between precipitates on the bottom and oil on the top was taken out and pipetted 10 times using 1 ml syringe. The entire volume of the liquid was added into an adsorption column provided with the kit and RNA was retained in the column while other components including DNA and protein were washed out by several buffer solutions. Total RNA was eluted in 50 μl RNase-free water. RNA-seq libraries were constructed by using the NEBNext Poly(A) mRNA Magnetic Isolation Module (NEB E7490) and NEBNext Ultra II Non-Directional RNA Second Strand Synthesis Module (NEB E6111). Briefly, mRNA was extracted by poly-A selected with magnetic beads with poly-T and transformed into cDNA by first and second strand synthesis. Newly synthesized cDNA was purified by AMPure XP beads (1:1) and eluted in 50 μL nucleotide-free water. Subsequent procedures were the same as ChIP-seq library construction described previously, except the sequencing depth of 20 million reads per library. RNA-seq libraries were sequenced by using the Illumina Hi-seq X Ten platform with pair-end reads of 150 bp.

### ChIP-Seq Data Processing

All ChIP-seq raw data in the fastq format were cleaned by removing the adaptor sequence. Cutadapt (version 1.16, http://cutadapt.readthedocs.io/en/stable/guide.html) was used for this step with the parameters -u 10 -u -15 -U 10 -U -15 -m 30. Cleaned reads were aligned to the mouse reference genome (mm10) using BWA (version 0.7.15, http://bio-bwa.sourceforge.net) with the default settings. Peaks calling was finished by MACS2 (version 2.1.1, https://github.com/taoliu/MACS) with the parameters--nomodel--keep-dup all -p 1E-10--broad--broad-cutoff 1E-10--extsize 147. Then, HOMER (http://homer.ucsd.edu/homer/index.html,v4.9,2-20-2017) annotatePeaks.pl was used to annotate ChIP-seq peaks compared to reference genome mm10. HOMER findMotifsGenome.pl was used to find out significant enriched motif. Replicates of ChIP-seq data were pooled for downstream analysis.

### RNA-Seq Data Processing

All RNA-seq raw data in the fastq format were cleaned by removing the adaptor sequence similar to ChIP-seq data processing. Cleaned reads were mapped to the mouse reference genome (mm10) using TopHat (version 2.1.1, http://ccb.jhu.edu/software/tophat/index.shtml) with the default settings. The gene expression level was calculated by Cufflinks (version 2.2.1, http://cole-trapnell-lab.github.io/cufflinks) and normalized by fragments per kilobase of bin per million mapped reads (FPKM). A differential gene expression analysis between tumor and normal tissue was performed using the R/Bioconductor package DESeq2 (version 1.26.0). Genes whose |log2FC| ≥ 1 and *p*-value < 0.05 were identified as differentially expressed genes (DEGs), and the gene ontology analysis was performed using DAVID (https://david.ncifcrf.gov).

### Comparison Between Different Histone Markers and Between Replicates

For comparison in the genome-wide scale, the genome was divided into massive 2 kb windows and the enrichment of histone modifications in each bin was used to calculate the correlation. For the comparison among enhancers, signal of modifications on each enhancer was normalized by reads per kilobase of bin per million mapped reads (RPKM) and such enrichment was used for calculating correlation.

### Identification of Typical and Super Enhancers

Enhancers were identified by the algorithm developed previously (Hnisz et al., 2013; Whyte et al., 2013). Briefly, significant distal H3K27ac peaks (peak boundary 1.5 kb or peak center 3 kb away from gene TSS) were identified, and the peaks whose distance was shorter than 12.5 kb were merged together as distal enhancers. The distal enhancers were ranked by a total signal of H3K27ac, and a plot was drawn to show the increased H3K27ac signal. Then, a tangent line with slope 1 was found for the curve, and the intersection point was determined as the infection point. Enhancers above this point were defined as super enhancers, and those below this point were defined as typical enhancers.

### Bioinformatic Analysis of Clinical Data

The clinical data analysis was performed by using the GEPIA (http://gepia.cancer-pku.cn/) and The Cancer Genome Atlas (TCGA) platforms. The expression level of *MMP10, CD300LF, MEFV, FOSL1, NLRP3, BFSP1, KLF7, E2F8, CTSC, FHL2, MAPK6, STAB1,* and *VAV1* in the patient’s HNSCC and precancerous tissues were calculated using the GEPIA platform. The correlation analysis was calculated using the HNSC dataset loading from the TCGA platform. DESeq2 (version 1.26.0) was used to screen for DEGs.

## Ethics Approval and Consent to Participate

All the animal operations were performed following the laboratory animal guidelines of Wuhan University and were approved by the Animal Experimentations Ethics Committee of Wuhan University (Protocol NO. 14110B). No patient study was involved and the consent to participate is not applicable.

## Results

### Global Gene Expression Profiling of HNSCC Tumors

To study the dynamic change of active enhancers during HNSCC transformation, we utilized a common-used HNSCC mouse model, a transgenic mouse with a combined *Tgfbr1/Pten* knockout (*K14-Cre* ^ERtam+/−^; *Tgfbr1* ^flox/flox^; *Pten* ^flox/flox^). With tamoxifen treatment in head and neck epithelium, the mice developed tumors within the head–neck region (Bian et al., 2012). Then normal skin and tumor tissues in head-neck region from three mice were collected and samples were prepared for RNA-Seq and ChIP-Seq analyses.

**FIGURE 1:**
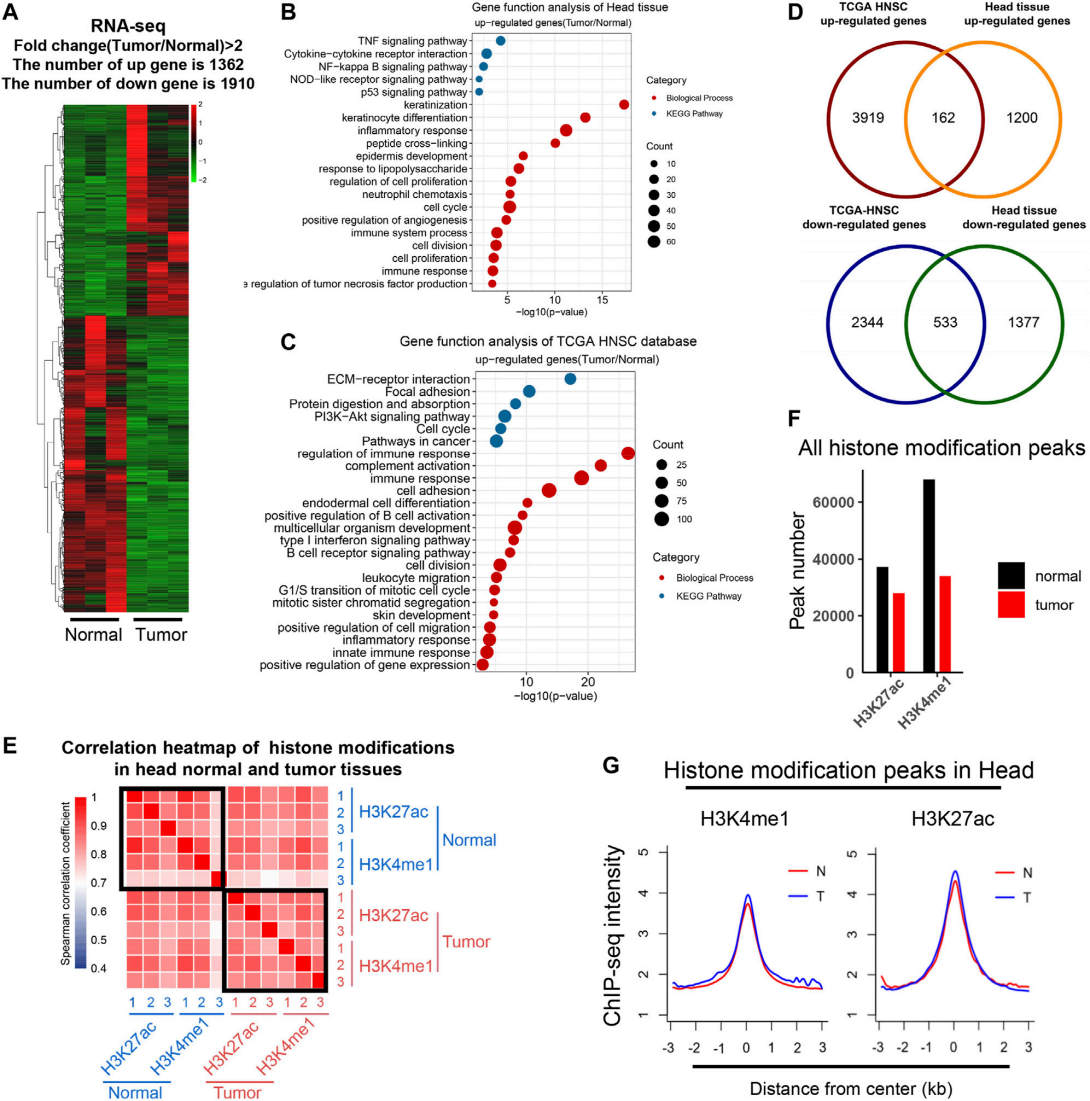
Transcriptomic and epigenomic profiling in an HNSCC model. **(A)** Heat map showed the differential gene expression (FPKM) between normal and tumor tissues; upregulated (red) and downregulated (green) genes (*n* = 3, Fold change ≥2). **(B and C)** Bubble plots showed biological process (red) and the KEGG pathway (blue) enrichment analysis of upregulated genes in mouse head tissues **(B)** and the TCGA HNSC database **(C)**, respectively. Items were ordered by the *p* value; the size of the dot represented the enriched gene count. **(D)** Venn diagrams showed the number of overlapped upregulated **(up)** and downregulated DEGs **(bottom)** between the head tissue and the TCGA HNSC database. **(E)** Heat map representing correlations based on H3K4me1 and H3K27ac occupancy on mouse genome. Correlations were calculated by the Spearman correlation coefficient. **(F)** Bar plot showed the number of significant peaks of H3K4me1 and H3K27ac in normal and tumor tissues. **(G)** Metagene plot of mean ChIP-seq signal of H3K4me1 and H3K27ac across the individual peaks of each mark. Metagene analysis was centered on the middle of peaks and 6 kb around peak centers are displayed (3 kb upstream and 3 kb downstream). The ChIP-seq data of each histone marks were merged from three individual replicates.

Analysis of DEGs revealed 1,362 upregulated and 1910 downregulated genes in tumor compared with normal tissues (fold change >2) (Figure 1A; Supplementary Table 1). The functional analysis of upregulated DEGs showed that genes involved in inflammatory response and cancer are enriched, such as TNF and p53 signaling pathways (Figure 1B). We downloaded data from TCGA HNSCC tissues and analyzed DEGs of tumor tissues (Supplementary Figure 1A; Supplementary Table 2). DEGs of our study were enriched in similar biological pathways to TCGA DEGs (Figures 1B,C; Supplementary Figure 1A–C; Supplementary Table 3). In comparison, 162 genes were overlapped for upregulated DEGs and 533 for downregulated DEGs (Figure 1D).

### Epigenomic Profiling of Histone Modifications in HNSCC Skin Tumors

To study the dynamic epigenetic state in HNSCC, we performed ChIP-Seq analyses of H3K27ac and H3K4me1 in control and tumor tissues. A correlation analysis of chromatin regions was performed to evaluate the consistency of all samples (Figure 1E). H3K4me1 and H3K27ac are marks for active enhancers, and the correlation among these samples was quite high, indicating our sequencing data were reliable. Peak numbers of H3K27ac and H3K4me1 decreased in tumor tissues, but the peak density did not change (Figures 1F,G). Chromatin distribution of H3K4me1 and H3K27ac peaks were mainly in intergenic and intron regions as expected (Supplementary Figure 1D). These indicated that our ChIP-Seq analyses of H3K4me1 and H3K27ac are consistent with the previously reported cases.

### Identification of Tumor-specific Active Enhancers

**FIGURE 2:**
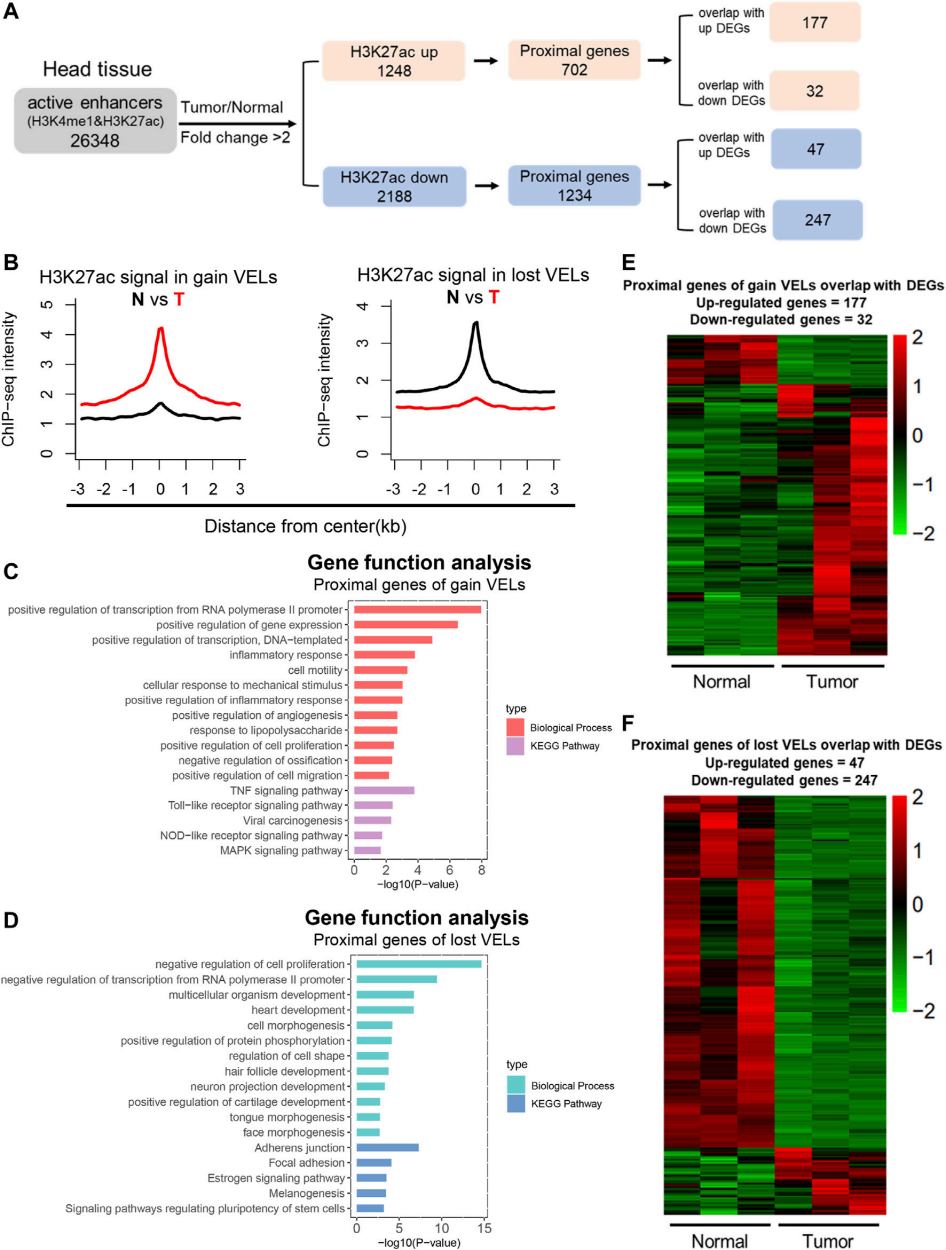
Identification of variant enhancer loci in HNSCC. **(A)** A sketch map to show the pipeline of enhancer analysis. Active enhancers were defined by H3K27ac and H3K4me1 and 2 kb away from TSS. **(B)** Aggregate plots comparing the mean H3K27ac signal (RPKM) across gain VELs and lost VELs in normal and tumor tissues. **(C and D)** Bar plots showed biological process and the KEGG pathway enrichment analysis of proximal genes for gain VELs and lost VELs, respectively. Items were ordered by the *p* value. **(E and F)** Heat maps showed the expression level of proximal genes of VELs overlapped with DEGs. (*n* = 3).

To identify variant enhancer loci (VELs) in HNSCC, we first identified active enhancers with H3K4me1 and H3K27ac peaks in normal and tumor tissues, respectively; then, we compared tumor and normal tissues and identified 1,248 gain VELs with H3K27ac upregulation in tumor (tumor/normal fold change ≥2), and 2,188 lost VELs with H3K27ac reduction (tumor/normal fold change ≤0.5). Next, the proximal genes to the above VELs were identified and overlapped with the above DEGs. Eventually, we identified 177 gain VELs with upregulated DEGs and 247 lost VELs with downregulated DEGs (Figure 2A; Supplementary Table 4). H3K27ac density on the above gain and lost VELs was shown (Figure 2B), and the signals of H3K4me1 and H3K27ac on gain VELs was significantly increased (Supplementary Figure 1E). The proximal genes of gain VELs were enriched in transcription regulation and cancer-related and inflammatory pathways (Figure 2C), and those of lost VELs were enriched in pathways of negative regulation of transcription and cell proliferation, as well as the development process (Figure 2D). Heatmaps for the expression of overlapped genes between VELs and DEGs were shown in Figures 2E,F. Our results indicated that the identified VELs were tightly associated with the gain of cancer features and loss of normal tissue features.

### Tumor-Specific Super Enhancers in Tumor Tissues

**FIGURE 3:**
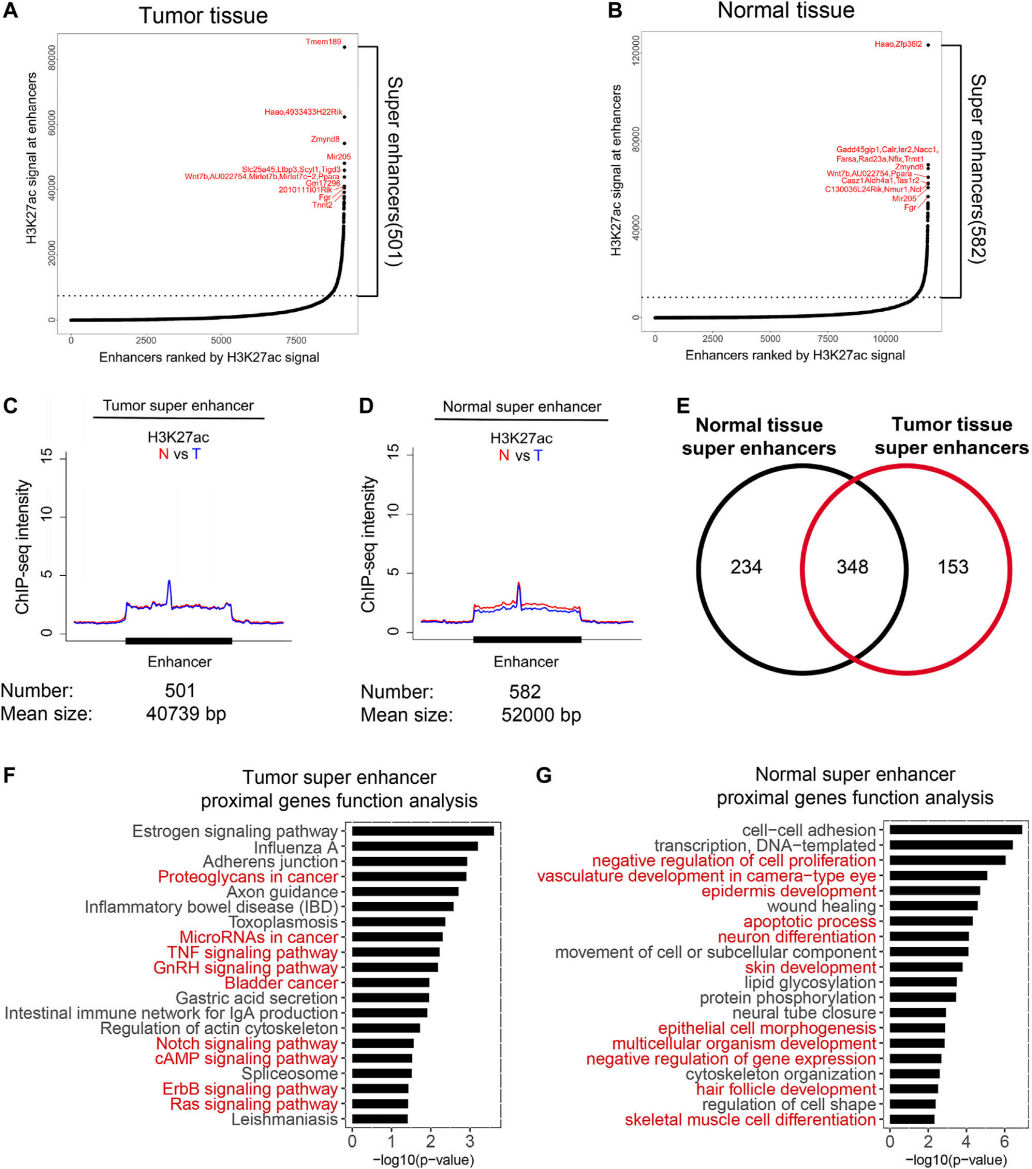
Identification of variant super enhancer loci in HNSCC. **(A and B)** Super enhancers in normal and tumor tissues were identified as described in the method section. The proximal genes of top 10 super-enhancers in normal and tumor tissues were marked. **(C and D)** Metagene analysis of mean H3K27ac ChIP-seq density across 501 super-enhancers in tumor **(C)** and 582 super-enhancers in normal tissues **(D)**. Metagene plot was centered on the enhancer region. The number and mean size of super-enhancers were shown. **(E)** Venn diagrams showed the overlapped super-enhancers number between normal and tumor tissues. **(F)** Gene function analysis of the proximal genes for tumor super-enhancers. Signaling pathways associated with cancer were highlighted in red. **(G)** Gene function analysis of the proximal genes for normal super-enhancers. Red markers represented signaling pathways associated with development processes were highlighted in red.

Super enhancers are considered as specific signatures for cell identity (Hnisz et al., 2013; Pott and Lieb, 2015). To reveal specific super enhancers of HNSCC, we analyzed super enhancers as previously described, and labeled them with their proximal genes (Figures 3A,B; Supplementary Table 5). It was hypothesized that the gain of super enhancers is one of the common features of tumor cells (Hnisz et al., 2013; Lovén et al., 2013). Surprisingly, the average H3K27ac intensity of super enhancers of tumor tissues showed no difference in normal and tumor tissues; however, super enhancers of normal tissues showed a slightly higher H3K27ac intensity in normal tissues. These results indicated that the average activity of the identified super enhancers in our model did not increase in tumor tissues. The total numbers of super enhancers in normal and tumor tissues were close and 348 were overlapped. We then analyzed their functions *via* proximal genes of super enhancers. Super enhancers of tumor tissues were enriched in genes regulating cancer-related pathways (Figure 3F), while those of normal tissues were enriched in development processes (Figure 3G). These indicated that the identified super enhancers in tumor tissue, especially the variant super enhancer loci (VSELs), play important roles in HNSCC.

### Transcription Factor AP-1 Is Widely Activated in Multiple Cancer Types

**FIGURE 4:**
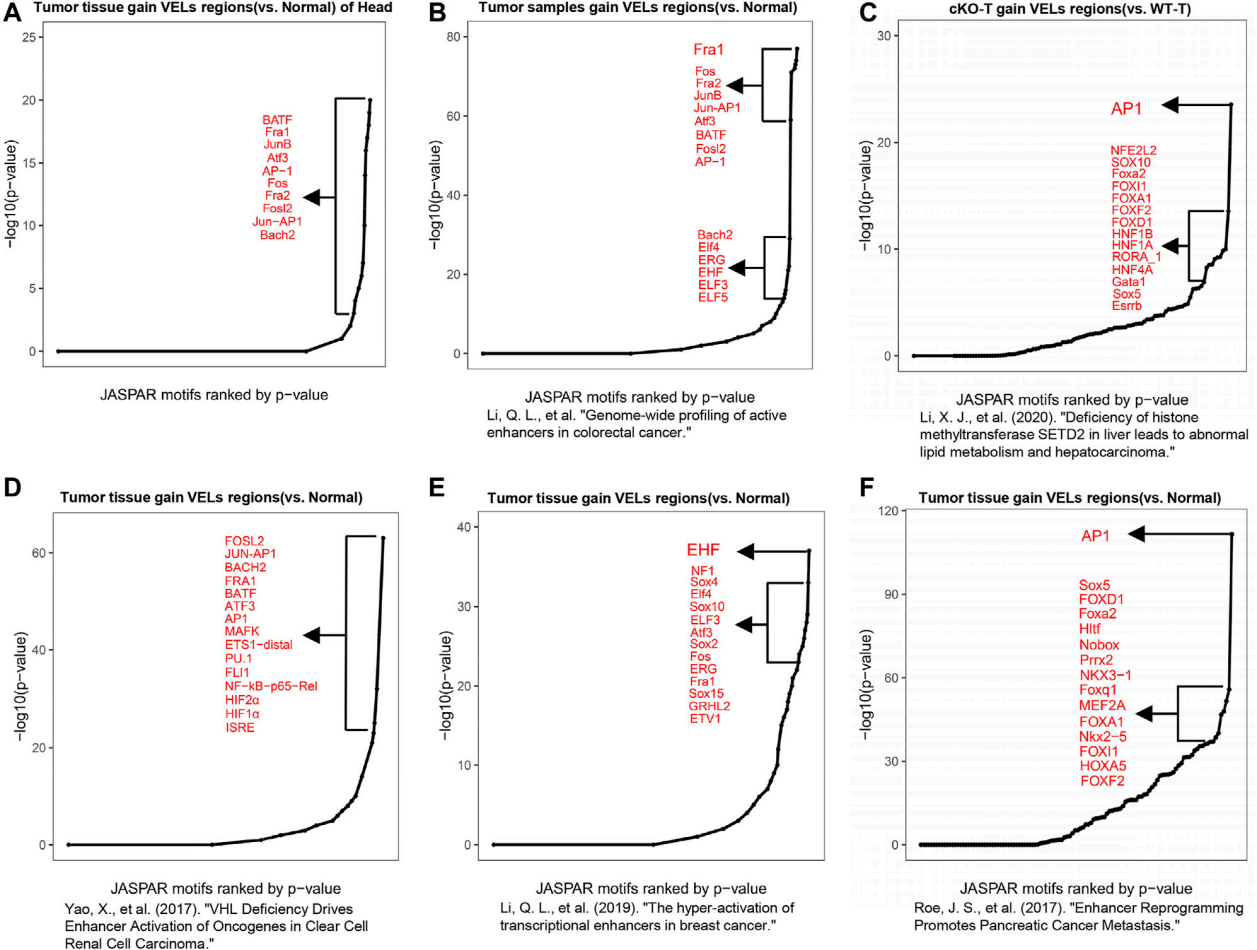
Motif analysis predicted the roles of AP-1 in cancer. **(A)** Motif analysis with DNA sequence information of gain VELs in tumor predicted functional TFs and a curve was drawn according to *p* values of TFs. The top 10 TFs were listed. **(B–F)** Motif analyses to predict functional TFs with published H3K27ac ChIP-Seq data in multiple cancers, including colorectal cancer **(B)**, hepatocarcinoma **(C)**, clear cell renal cell carcinoma **(D)**, breast cancer **(E)**, and pancreatic cancer **(F)**.

Motif analysis using VEL information can be used to predict important TFs involved in biological processes (Shlyueva et al., 2014). We analyzed the gain VELs in HNSCC and found that the predicted top transcription factors were either members of the AP-1 TF family or their binding partners, such as *cJun*, *JunB*, *Fos*, *BATF*, and *ATF3* (Figure 4A; Supplementary Table 6). Based on the literatures and our experience, we found that AP-1 emerged to be one of the top TFs predicted in multiple cancers. We analyzed data from our own lab and other groups, including colorectal cancer, hepatocarcinoma, kidney cancer, breast cancer, and pancreatic cancer (Roe et al., 2017; Yao et al., 2017; Li et al., 2019; Li et al., 2020; Li et al., 2021). The results indicated that AP-1 family members were among the top TFs predicted with the above datasets (Figures 4B–F), implying that AP-1 plays critical roles in multiple cancers.

### Transcription Program Targeted by AP-1 in HNSCC

**FIGURE 5:**
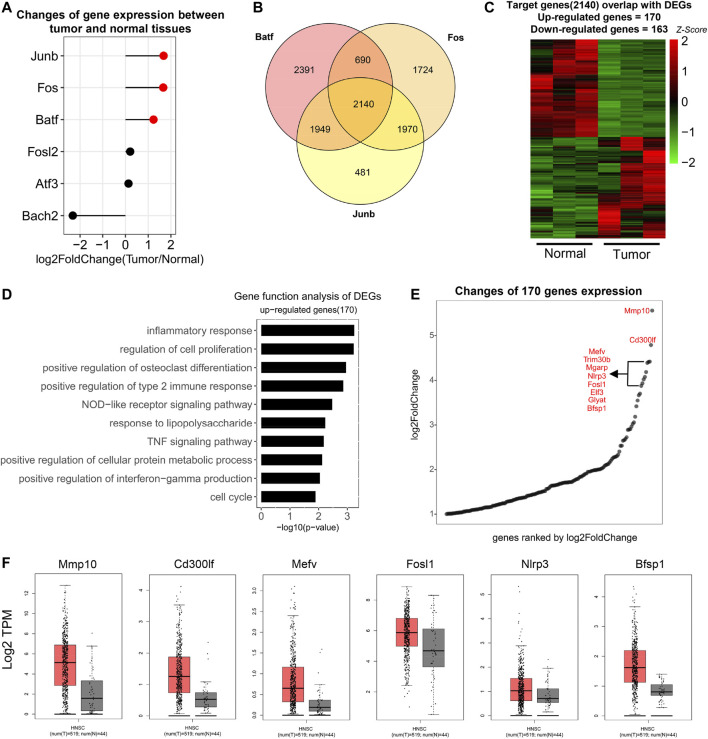
Target genes controlled by AP-1 in HNSCC. **(A)** Matchstick plot showed the expression change folds of AP-1 family TFs between tumor and normal tissues. TFs highly expressed in tumor were highlighted in red. Cutoff: log2Foldchange ≥1. **(B)** Venn diagrams to show the number of overlapped genes among Batf, Fos, and Junb target genes. Target genes were derived from Cistrome database. (http://cistrome.org/db/#/). **(C)** Heat map to show the expression of overlapped genes between AP-1 target genes (2,140) and HNSCC DEGs. 170 genes were upregulated and 163 genes were downregulated in tumor (*n* = 3). **(D)** Gene function analysis of upregulated AP-1 target genes (170). Items were ordered by the *p* value. **(E)** Point diagram to show the fold-change (tumor/normal) of 170 upregulated AP-1 target genes. Genes were ranked by log2 fold-change and the top 10 genes were listed. **(F)** Boxplots to show the expression (log2 TPM) of the top 10 genes in Figure 4E in HNSCC tumor and normal tissues in the TCGA database. Only the genes with significant change were displayed.

To further explore the role of AP-1 in HNSCC, we first analyzed the expression of AP-1 family members in normal and tumor tissues. Among the predicted AP-1 members, *Junb*, *Fos,* and *Batf* were expressed higher in tumor tissues (Figure 5A). We then downloaded ChIP-Seq datasets of the above three proteins from the Cistrome database (http://cistrome.org/db/#/) and predicted their target genes by identifying proximal genes to their binding sites. Their target genes were then overlapped and 2,140 genes targeted by all 3 TFs were used for further study (Figure 5B). We overlapped the above target genes with DEGs and identified 170 upregulated and 163 downregulated genes (Figure 5C). These genes were probably targeted by AP-1 family members in the current HNSCC model. The gene function analysis indicated that they were enriched in pathways related with inflammation, cell proliferation, and protein metabolic process, very similar to the previous analysis about all upregulated DEGs (Figures 2C, 5D), further supporting that AP-1 family members are critical in HNSCC. We then took the top 10 DEGs targeted by AP-1 and studied their expression in HNSCC patients using TCGA datasets. The result indicated that 6 genes are highly expressed in HNSCC patient tissues, including *Mmp10*, *Cd300lf*, *Mefv*, *Fosl1*, *Nlrp3,* and *Bfsp1*.

### Coordination of AP-1 and Histone Modifications in Transcriptional Programming

**FIGURE 6:**
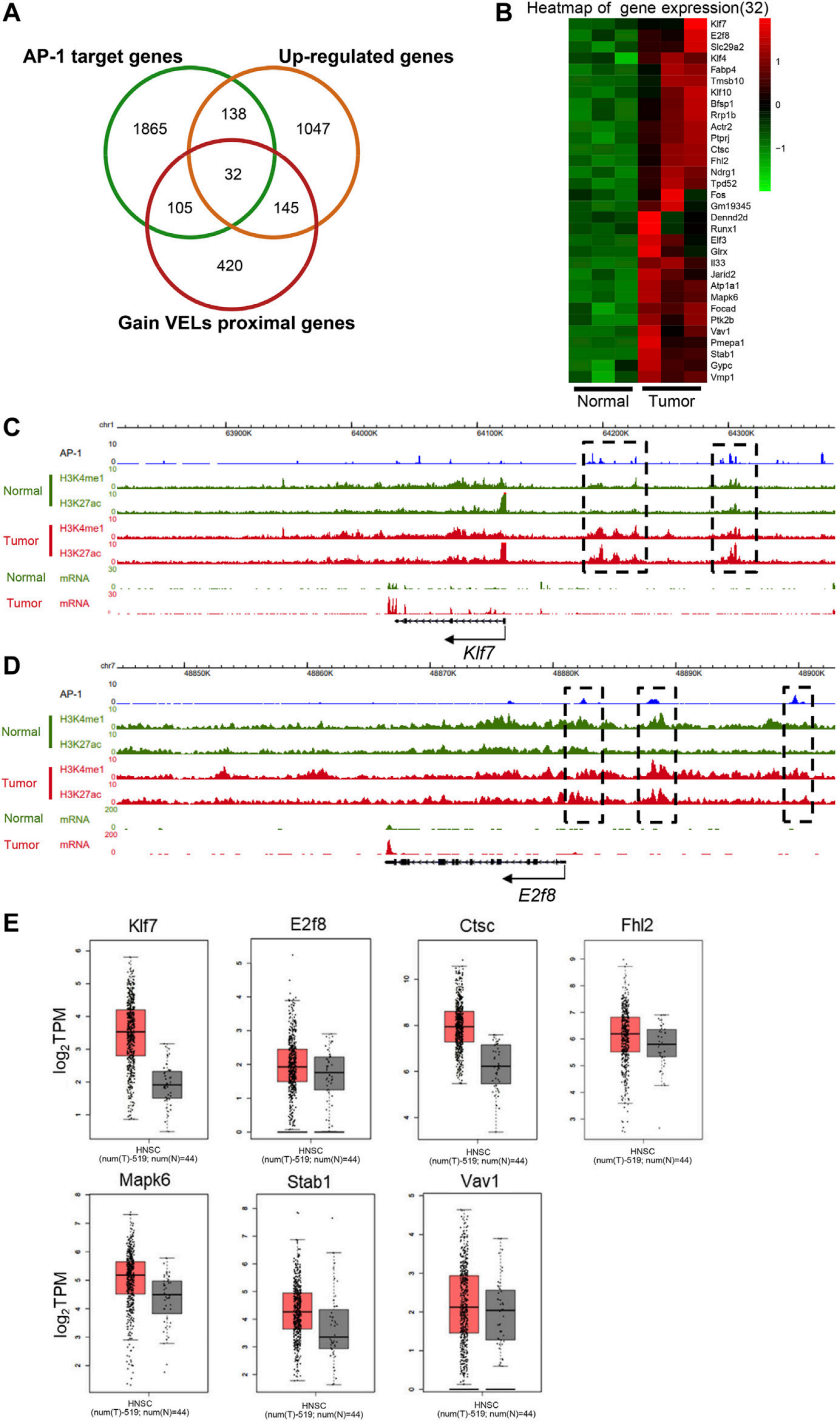
Coordination of AP-1 and enhancer histone modifications on transcription regulation. **(A)** Venn diagram to show the overlapped genes numbers between AP-1 target genes, upregulated DEGs and gain VEL proximal genes. **(B)** Heat map to show the gene expression of the 32 overlapped target genes in Figure A. **(C and D)** The genome browser view showed H3K27ac and H3K4me1 signals, and mRNA expression in normal and tumor tissues of *Klf7* (C) and *E2f8* (D) genes. The black boxes highlighted the identified VELs. **(E)** Box plots to show the expression (log2 TPM) of the identified overlapped genes of Figure 6B in tumor and normal tissues in TCGA database. *Bfsp1* was shown in Figure 5F.

To investigate the interplay of AP-1 transcription factors and epigenetic modifications, we overlapped AP-1-targeted DEGs with proximal genes of gain VELs (Figures 6A,B). Totally 32 genes were identified and their expression levels were shown in Figure 6B. The view of the UCSC browser indicated that H3K4me1 and H3K27ac peaks of a portion of genes were nicely correlated with AP-1 binding sites on gene bodies and enhancers (Figures 6C,D; Supplementary Figure 2A–G). The black boxes highlighted AP-1 binding sites and their corresponding histone modifications. For some genes, such as *Klf7*, both H3K4me1 and H3K27ac increased on the AP-1 enhancers in tumor tissues (Figure 6C); for others, such as E2f8, H3K4me1 did not change obviously while H3K27ac significantly increased (Figure 6D). A portion of the 32 genes also have higher expression in HNSCC patient tissues according to TCGA data, including *Klf7*, *E2f8*, *Ctsc*, *Fhl2*, *Mapk6*, *Stab1*, *Vav1*, and *Bfsp1* (Figures 5F, 6E). These suggested that AP-1 and histone modifications probably regulate target gene expression synergistically in HNSCC.

## Discussion

Epigenetic regulation is critical for tumorigenesis and metastasis (Castilho et al., 2017; Leemans et al., 2018; Gazdzicka et al., 2020). Activation of enhancers for oncogenes and repression of enhancers for tumor suppressors are important for tumor cell transformation. In the current study, we investigated the genome-wide distribution of active enhancers in an HNSCC model and identified 1,248 gain VELs and 2,188 lost VELs, as well as 153 gain VSELs and 234 lost VSELs. Function analysis of their proximal genes indicated they are tightly related with HNSCC. Moreover, we identified AP-1 as one of the critical oncogenic transcription factors in HNSCC, which also plays important roles in many other types of cancer. Further analysis identified 170 upregulated DEGs as potential AP-1 target genes in the HNSCC model, whose functions are enriched in inflammation and the cell proliferation process. An integrated analysis with VEL information further identified 32 AP-1 target genes with coordinated changes of histone modifications. Among these, quite a few genes are highly expressed in HNSCC patient tissues. All the above-identified VELS, VSELs, TFs, and target genes provide important information for HNSCC studies.

Unlike previous studies, the average H3K27ac level did not increase significantly on tumor super enhancers in the studied HNSCC model (Figures 3C,D), indicating that the average enhancer activity is not necessarily upregulated in all cancers. However, the functions of super enhancers in tumor tissues are still tightly related with cancer, suggesting super enhancers still can be considered as specific features of cell identity.

Previously, AP-1 was reported to be critical in multiple cancers; however, it is also tightly related with cell death and other processes. So, it was considered as a double-edged sword for cancer (Shaulian and Karin, 2002; Eferl and Wagner, 2003; Gross et al., 2007; Shaulian, 2010; Sun et al., 2018; Li et al., 2021). Our analyses with current HNSCC and other data support that AP-1 family members are among the most frequently activated TFs in multiple cancers, which govern the gene expression involved in inflammation and cell proliferation. AP-1 is often activated under stress conditions (Hayes et al., 2020), and it is probably critical to further investigate the underlying mechanisms in tumorigenesis and metastasis.

Our study not only revealed AP-1 as a critical TF for HNSCC but also identified its target genes by combining transcriptomics and epigenomics data. A large portion of the identified genes have been reported to be associated with HNSCC or multiple types of cancer. Thus, we have revealed new mechanisms for HNSCC tumorigenesis regulated by AP-1. Among the target genes, *Fhl2* was reported to contribute to tongue squamous cell carcinoma and other cancers (Nakazawa et al., 2016; Wang et al., 2016; Li et al., 2021); *Mapk6* is a Ser/Thr protein kinase and is related with oral squamous cell carcinoma and other cancers (Rai et al., 2004). *Klf7* is an oncogenic TF related with lung adenocarcinoma and gastric cancer (Jiang et al., 2017; Niu et al., 2020); *E2f8* is a TF-regulating cell cycle and involved in cervical cancer and hepatocellular carcinoma (Eoh et al., 2020; Kim et al., 2020); *Vav1* is associated with esophageal squamous cell carcinoma and indicates poor prognosis, and it is involved in the JNK/SAPK cascade (Möller et al., 2001; Zhu et al., 2017). Further studies about these genes will increase our knowledge about HNSCC.

## Data Availability

The original contributions presented in the study are publicly available. This data can be found here: GEO database, Acc. NO.GSE172324.
