# Supplementary material for: Genome-Wide Enhancer Analysis Reveals the Role of AP-1 Transcription Factor in Head and Neck Squamous Cell Carcinoma
Source: Front Mol Biosci. 2021 Aug 2;8:701531. doi: 10.3389/fmolb.2021.701531 (PMC8365880; doi:10.3389/fmolb.2021.701531)
Supplement: Supplementary file 1 [file DataSheet1.PDF]

Sup. Fig. 1

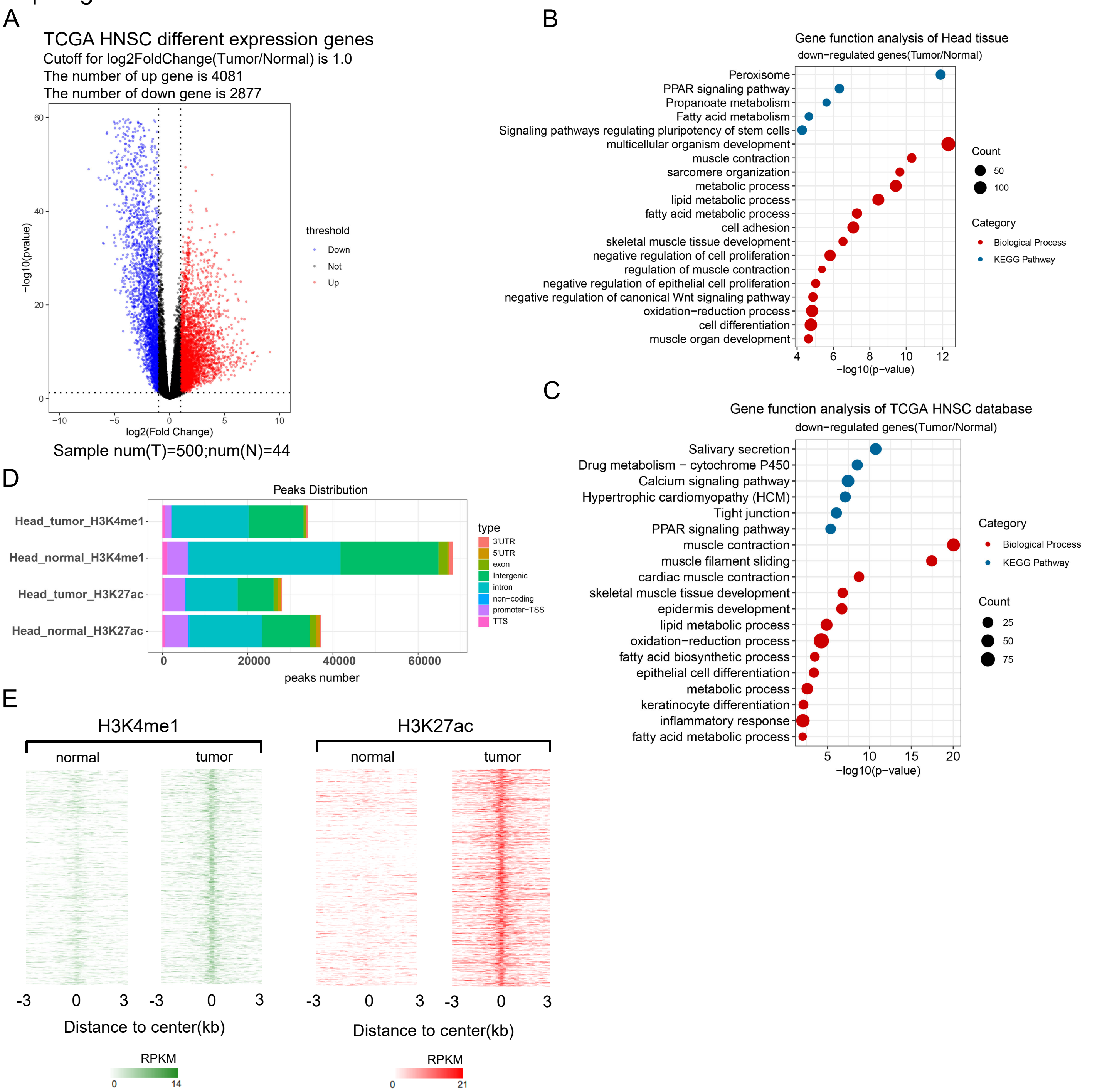

Sup. Fig. 1 Transcriptomic and epigenomic profiling in a HNSCC model. (A) Volcano plot of the log2 Foldchange (tumor/normal) gene expression versus the −log10 (p value) for TCGA HNSC database all human genes between tumor and native tissues. Red dots represented tumor up-regulated genes, blue dots represented native tissue up-regulated genes and black dots for genes not changed. (FDR < 0.05 and fold change < −2 or fold change > 2, respectively). **(B&C)** Bubble Plot showed the Biological process(red) and KEGG pathway(blue) enrichment analysis of down-regulated genes in Head tissue and TCGA HNSC database respectively, items were ordered by p value, the size of the dot represented the enriched gene count. **(D)** The distribution of H3K4me1 and H3K27ac peaks of tumor and normal tissue samples in different genome elements, calculated by HOMER module *annotatePeaks.pl*. Promoter-TSS,"-1kb to +100bp" of transcription start sites.TTS,"-100bp to +1kb" of transcription termination sites. **(E)** Heatmaps showed H3K4me1 and H3K27ac signals(RPKM) within GAIN VELs regions in tumor and normal tissues. All rows are centered on the peaks center ± 3kb.

Sup. Fig. 2

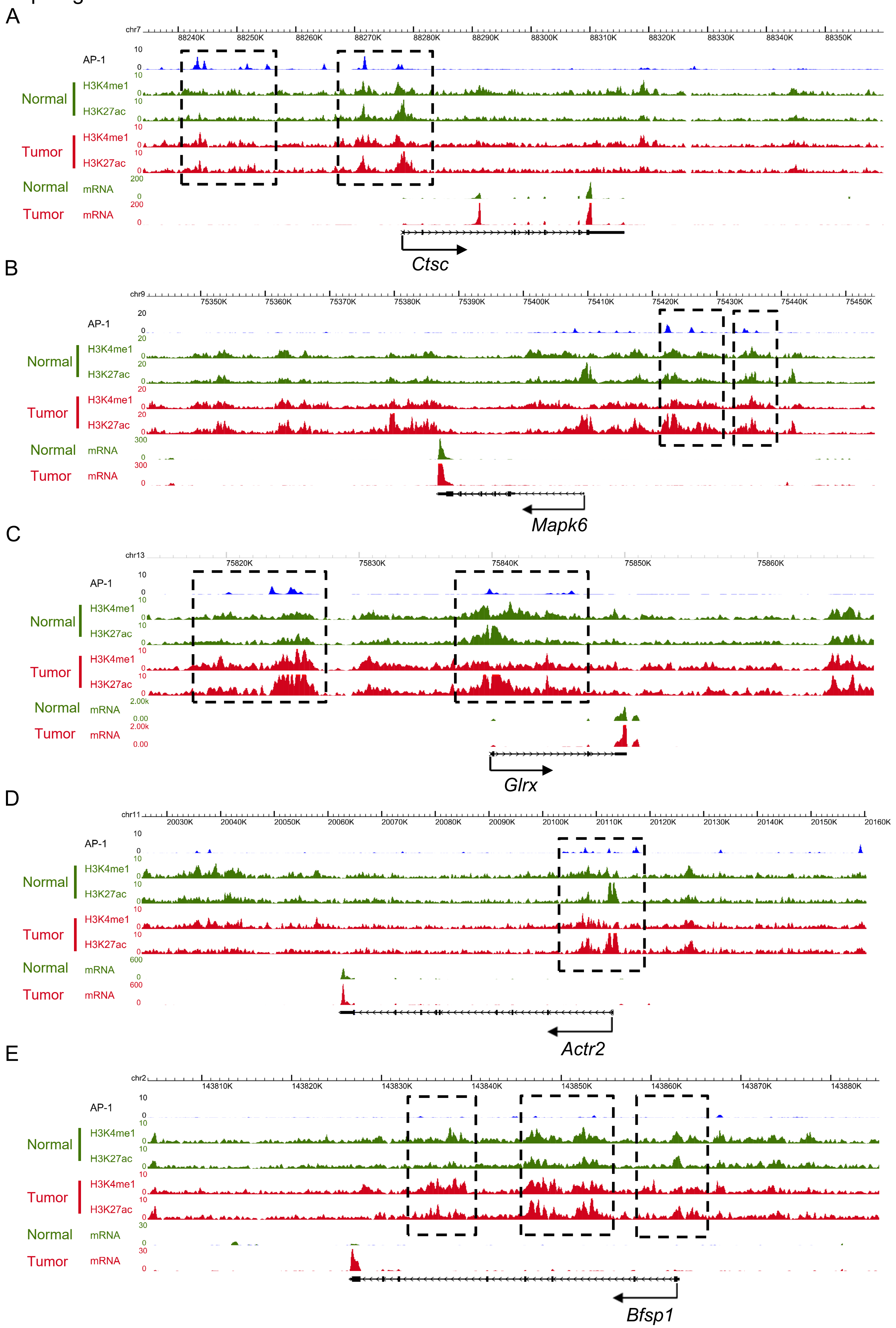
A
chr7
88240K
88250K
88260K
88270K
88280K
88290K
88300K
88310K
88320K
88330K
88340K
88350K
AP-1
Normal
H3K4me1
H3K27ac
Tumor
H3K4me1
H3K27ac
Normal mRNA
Tumor mRNA
Ctsc
B
chr9
75350K
75360K
75370K
75380K
75390K
75400K
75410K
75420K
75430K
75440K
75450K
AP-1
Normal
H3K4me1
H3K27ac
Tumor
H3K4me1
H3K27ac
Normal mRNA
Tumor mRNA
Mapk6
C
chr13
75820K
75830K
75840K
75850K
75860K
AP-1
Normal
H3K4me1
H3K27ac
Tumor
H3K4me1
H3K27ac
Normal mRNA
Tumor mRNA
2.00k
0.00
Glrx
D
chr11
20030K
20040K
20050K
20060K
20070K
20080K
20090K
20100K
20110K
20120K
20130K
20140K
20150K
20160K
AP-1
Normal
H3K4me1
H3K27ac
Tumor
H3K4me1
H3K27ac
Normal mRNA
Tumor mRNA
Actr2
E
chr2
143810K
143820K
143830K
143840K
143850K
143860K
143870K
143880K
AP-1
Normal
H3K4me1
H3K27ac
Tumor
H3K4me1
H3K27ac
Normal mRNA
Tumor mRNA
Bfsp1

Sup. Fig. 2

F

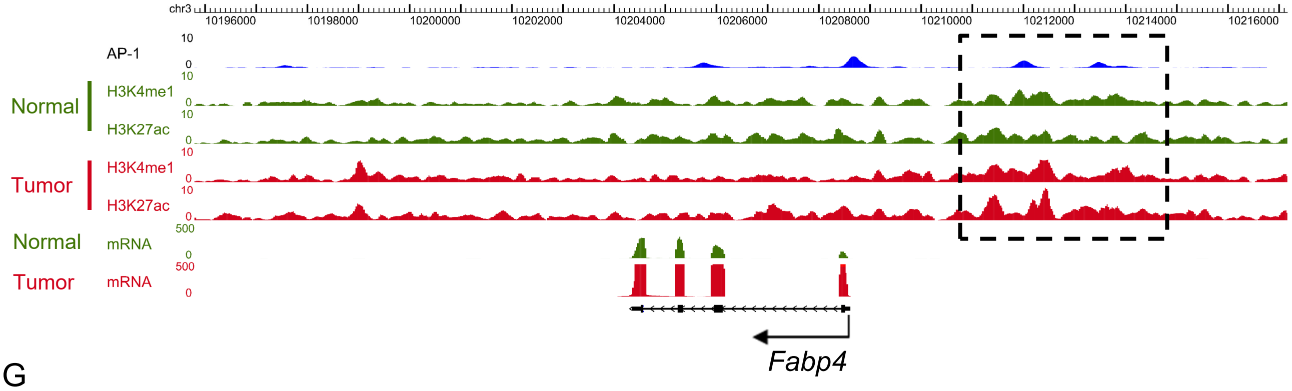

G

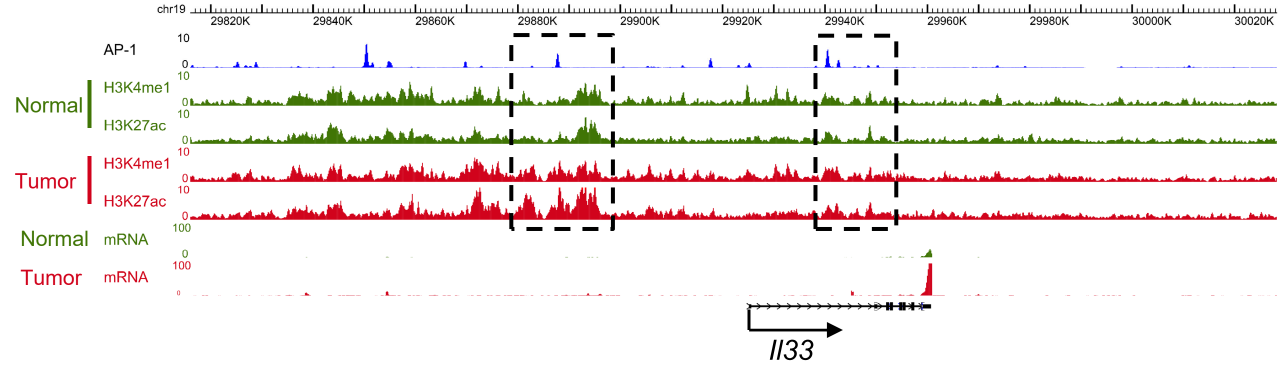

Sup. Fig. 2 UCSC browser views for representative AP-1 target genes. The genome browser view showed the ChIP-seq density of H3K27ac, H3K4me1, and RNA-seq signal in both normal tissue and tumor located in *Ctsc* (A), *Mapk6* (B), *Glrx* (C), *Actr2* (D), *Bfsp1* (E), *Fabp4* (F), *Il33* (G) locus, and the black box represented the possible VELs loci.
